# Honey Bees Inspired Optimization Method: The Bees Algorithm

**DOI:** 10.3390/insects4040646

**Published:** 2013-11-06

**Authors:** Baris Yuce, Michael S. Packianather, Ernesto Mastrocinque, Duc Truong Pham, Alfredo Lambiase

**Affiliations:** 1 Institute of Sustainable Engineering, School of Engineering, Cardiff University, Queen’s Buildings, The Parade, Cardiff CF24 3AA, UK; 2 Institute of Mechanical and Manufacturing Engineering, School of Engineering, Cardiff University, Queen’s Buildings, The Parade, Cardiff CF24 3AA, UK; E-Mail: PackianatherMS@cardiff.ac.uk; 3 Department of Industrial Engineering, University of Salerno, Via Ponte don Melillo 1, Fisciano 80046, Italy; E-Mails: emastrocinque@unisa.it (E.M.); lambiase@unisa.it (A.L.); 4 School of Mechanical Engineering, University of Birmingham, Edgbaston, Birmingham B15 2TT, UK; E-Mail: d.t.pham@bham.ac.uk

**Keywords:** honey bee, foraging behavior, waggle dance, bees algorithm, swarm intelligence, swarm-based optimization, adaptive neighborhood search, site abandonment, random search

## Abstract

Optimization algorithms are search methods where the goal is to find an optimal solution to a problem, in order to satisfy one or more objective functions, possibly subject to a set of constraints. Studies of social animals and social insects have resulted in a number of computational models of swarm intelligence. Within these swarms their collective behavior is usually very complex. The collective behavior of a swarm of social organisms emerges from the behaviors of the individuals of that swarm. Researchers have developed computational optimization methods based on biology such as Genetic Algorithms, Particle Swarm Optimization, and Ant Colony. The aim of this paper is to describe an optimization algorithm called the Bees Algorithm, inspired from the natural foraging behavior of honey bees, to find the optimal solution. The algorithm performs both an exploitative neighborhood search combined with random explorative search. In this paper, after an explanation of the natural foraging behavior of honey bees, the basic Bees Algorithm and its improved versions are described and are implemented in order to optimize several benchmark functions, and the results are compared with those obtained with different optimization algorithms. The results show that the Bees Algorithm offering some advantage over other optimization methods according to the nature of the problem.

## 1. Introduction

Swarm Intelligence (SI) is defined as the collective problem-solving capabilities of social animals [1]. SI is the direct result of self-organization in which the interactions of lower-level components create a global-level dynamic structure that may be regarded as intelligence [2]. These lower level interactions are guided by a simple set of rules that individuals of the colony follow without any knowledge of its global effects [2]. Individuals in the colony only have local-level information about their environment. Using direct and/or indirect methods of communication, local-level interactions affect the global organization of the colony [2].

Self-organization is created by four elements as were suggested by Bonabeau *et al*. [1]. Positive feedback is defined as the first rule of self-organization. It is basically a set of simple rules that help to generate the complex structure. Recruitment of honey bees to a promising flower patch is one of the examples of this procedure [2]. The second element of self-organization is negative feedback, which reduces the effects of positive feedback and helps to create a counterbalancing mechanism. The number of limited foragers is an example of negative feedback [2]. Randomness is the third element in self-organization. It adds an uncertainty factor to the system and enables the colonies to discover new solutions for their most challenging problems (food sources, nest sites, *etc.*). Finally, there are multiple interactions between individuals. There should be a minimum number of individuals who are capable of interacting with each other to turn their independent local-level activities into one interconnected living organism [2]. As a result of combination of these elements, a decentralized structure is created. In this structure there is no central control even though there seems to be one. A hierarchical structure is used only for dividing up the necessary duties; there is no control over individuals but over instincts. This creates dynamic and efficient structures that help the colony to survive despite many challenges [2].

There are many different species of animal that benefit from similar procedures that enable them to survive and to create new and better generations. Honey bees, ants, flocks of birds and shoals of fish are some of the examples of this efficient system in which individuals find safety and food. Moreover, even some other complex life forms follow similar simple rules to benefit from each other’s strength [2]. To some extent, even the human body can be regarded as a self-organized system. All cells in the body benefit from each other’s strength and share the duties of overcoming the challenges, which are often lethal for an individual cell [2]. 

Swarm-based optimization algorithms (SOAs) mimic nature’s methods to drive a search towards the optimal solution. A key difference between SOAs and direct search algorithms such as hill climbing and random walk is that SOAs use a population of solutions for every iteration instead of a single solution. As a population of solutions is processed as iteration, the outcome of each iteration is also a population of solutions [2]. If an optimization problem has a single optimum, SOA population members can be expected to converge to that optimum solution. However, if an optimization problem has multiple optimal solutions, an SOA can be used to capture them in its final population. SOAs include Evolutionary Algorithms [3] (*i.e.*, the Genetic Algorithm), the Particle Swarm Optimization (PSO) [4] Artificial Bee Colony (ABC) Optimization [5,6] the Ant Colony Optimization (ACO) [7]. Common to all population-based search methods is a strategy that generates variations of the solution being sought. Some search methods use a greedy criterion to decide which generated solution to retain. Such a criterion would mean accepting a new solution if and only if it increases the value of the objective function.

The aim of this paper is to describe an optimization algorithm called the Bees Algorithm, introduced by Pham [8], inspired from the natural foraging behavior of honey bees, to find the optimal solution. The algorithm performs both an exploitative neighborhood search combined with random explorative search. The BA has been successfully applied on several optimization problems as multi-objective optimization [9], neural network training [10], manufacturing cell formation [11], job shop scheduling for a machine [12], data clustering [13], optimizing the design of mechanical components [14], image analysis [15], and supply chain optimization [16].

In this paper, after an explanation of the natural foraging behavior of honey bees, the basic Bees Algorithm and its improved versions are described and are implemented in order to optimize several benchmark functions, and the results are compared with those obtained with different optimization algorithms. The paper is organized as follows: Swarm-optimization algorithms is given in Section 2; the description of the foraging behavior of honey bees is given is Section 3; the description of the Bees algorithm is given in Section 4; an improved version of the Bees Algorithm is given in Section 5; the experimental results and discussion are given in Section 6; the conclusions are given in Section 7.

## 2. Swarm-Optimization Algorithms

Swarm Optimization Algorithms (SOAs) mimic the collective exploration strategy of the swarms in the nature on optimization problems [2]. These algorithms utilize a population based approach to the problems. This group of algorithms is known as population based stochastic algorithms [15]. The most famous swarm algorithms are the Evolutionary Algorithms (EAs), Particle Swarm Optimization (PSO), Ant Colony Optimization (ACO) and The Bees-inspired Algorithm (BA) such as Artificial Bee colony and the Bees Algorithm.

### 2.1. Evolutionary Algorithms

Evolutionary Algorithms (EAs) are well-known SOAs, and inspired from the natural selection, mutation and recombination of the biological mechanism. Several forms of these algorithms have been introduced such as Evolutionary Strategy, Evolutionary Programming, Genetic Algorithm and so on. In EAs, the main strategy is to find the optimal points by utilizing the stochastic search operators such as natural selection, mutation and recombination to the population. The algorithms work with a random population of solutions. The algorithm efficiently exploits historical information to speculate on new search areas with improved performance [17]. When applied to optimization problems, the EA has the advantage of performing a global search [18].

### 2.2. Particle Swarm Optimization

Particle Swarm Optimization (PSO) utilizes the social behavior of the groups of population in nature such as animal herds or bird flocking, or schooling of fish. PSO consists of a population called swarm and each member of the swarm is called a particle [18]. The particles search the global optimum with a set velocity. Because the particles modify and update the position with respect to itself and its neighborhood, it has the capability to do both local and global searches [19]. 

### 2.3. Ant Colony Optimization

Ant Colony Optimization (ACO) was inspired by the pheromone-based strategy of ants foraging in nature. The foraging behavior of ants is based on finding the shortest path between source and their nests [20]. During the foraging process, ants leave their pheromone trails on the path when they return to their nest from the source, so the other members of the colony find the path by using the pheromone trails and pheromone level. If the selected path is the shortest path, then the pheromone level will be reinforced otherwise it will evaporate as time passes [21]. This behavior of ants inspired to implement one of the hard optimization problems called combinatorial optimization [22].

### 2.4. Bee-Inspired Algorithms

In nature, honey bees have several complicated behaviors such as mating, breeding and foraging. These behaviors have been mimicked for several honey bee based optimization algorithms.

One of the famous mating and breeding behavior of honey bees inspired algorithm is Marriage in Honey Bees Optimization (MBO). The algorithm starts from a single queen without family and passes on to the development of a colony with family having one or more queens. In the literature, several versions of MBO have been proposed such as Honey-Bees Mating Optimization (HBMO) [23], Fast Marriage in Honey Bees Optimization (FMHBO) [24] and The Honey-Bees Optimization (HBO) [25].

The other type of bee-inspired algorithms mimics the foraging behavior of the honey bees. These algorithms use standard evolutionary or random explorative search to locate promising locations. Then the algorithms utilize the exploitative search on the most promising locations to find the global optimum. The following algorithms were inspired from foraging behavior of honey bees; Bee System (BS), Bee Colony Optimization (BCO), Artificial Bee Colony (ABC) and The Bees Algorithm (BA). 

Bee System is an improved version of the Genetic Algorithm (GA) [26]. The main purpose of the algorithm is to improve local search while keeping the global search ability of GA. 

Bee Colony Optimization (BCO) was proposed to solve combinatorial optimization problems by [27]. BCO has two phases called forward pass and backward pass. A partial solution is generated in the forward pass stage with individual exploration and collective experience, which will then be employed at the backward pass stage. In the backward pass stage the probability information is utilized to make the decision whether to continue to explore the current solution in the next forward pass or to start the neighborhood of the new selected ones. The new one is determined using probabilistic techniques such as the roulette wheel selection. 

Artificial Bee Colony optimization (ABC) was proposed by Karaboga *et al.* [28,29,30]. The algorithm consists of the following bee groups: employed bees, onlooker bees and scout bees as in nature. Employed bees randomly explore and return to the hive with information about the landscape. This explorative search information is shared with onlooker bees. The onlooker bees evaluate this information with a probabilistic approach such as the roulette wheel method [28] to start a neighborhood search. Meanwhile, the scout bees perform a random search to carry out the exploitation. 

The Bees Algorithm was proposed by Pham *et al*. [7], which is very similar to the ABC in the sense of having local search and global search processes. However there is a difference between both algorithms during the neighborhood search process. As mentioned above, ABC has a probabilistic approach during the neighborhood stage; however the Bees Algorithm does not use any probability approach, but instead uses fitness evaluation to drive the search. In the following section the Bees Algorithm will be explained in detail.

## 3. The Foraging Behavior of Honey Bees

A colony of honey bees can exploit a large number of food sources in big fields and they can fly up to 11 km to exploit food sources [31,32]. The colony employs about one-quarter of its members as forager bees. The foraging process begins with searching out promising flower patches by scout bees. The colony keeps a percentage of the scout bees during the harvesting season. When the scout bees have found a flower patch, they will look further in hope of finding an even better one [32]. The scout bees search for the better patches randomly [33].

The scout bees inform their peers waiting in the hive as to the quality of the food source, based amongst other things, on sugar levels. The scout bees deposit their nectar and go to the dance floor in front of the hive to communicate to the other bees by performing their dance, known as the “waggle dance” [31].

### The Waggle Dance of Honey Bees

The waggle dance is named based on the wagging run (in which the dancers produce a loud buzzing sound by moving their bodies from side to side), which is used by the scout bees to communicate information about the food source to the rest of the colony. The scout bees provide the following information by means of the waggle dance: the quality of the food source, the distance of the source from the hive and the direction of the source [32,33].

**Figure 1:**
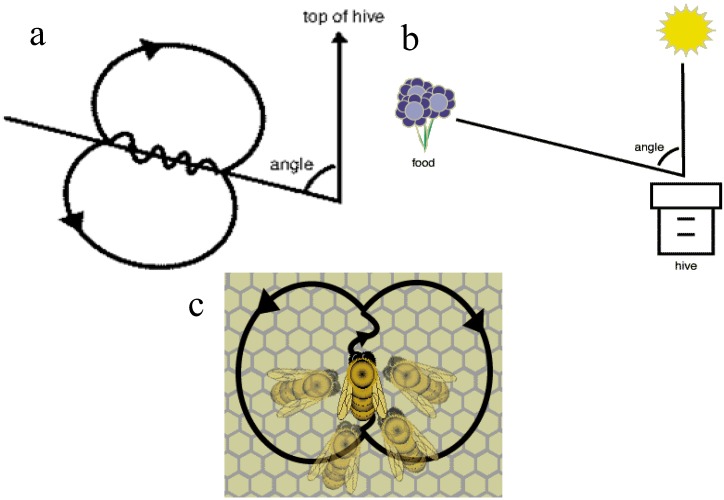
(**a**) Orientation of waggle dance with respect to the sun; (**b**) Orientation of waggle dance with respect to the food source, hive and sun; (**c**) The Waggle Dance and followers.

The waggle dance path has a figure of eight shape. Initially the scout bee vibrates its wing muscles which produces a loud buzz and runs in a straight line the direction which is related to the vertical on the hive and indicates the direction of the food source relative to the sun’s azimuth in the field, see Figure 1a,b [34]. The scout then circles back, alternating a left and a right return path [35]. The speed/duration of the dance indicates the distance to the food source; the frequency of the waggles in the dance and buzzing convey the quality of the source; see Figure 1c [34]. This information will influence the number of follower bees.

## 4. The Bees Algorithm

**Figure 2:**
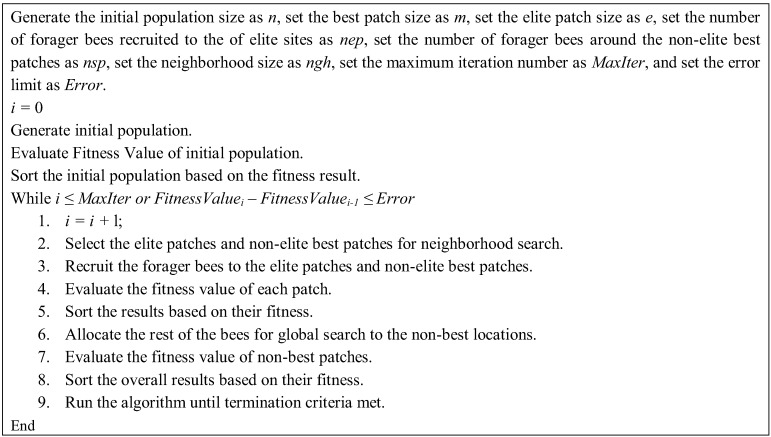
Pseudo-code of the basic Bees Algorithm.

**Figure 3:**
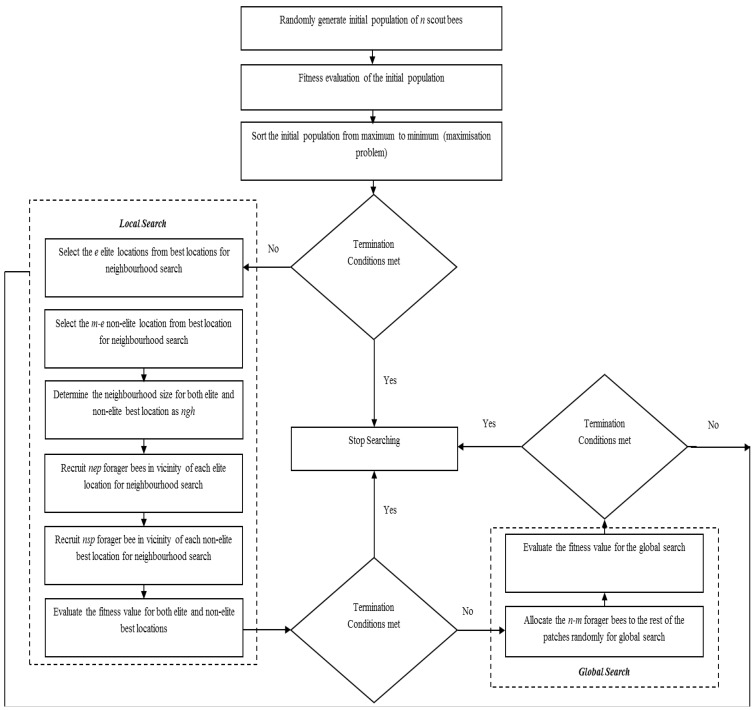
Flowchart of the basic Bees Algorithm.

The BA has both local and global search capability utilizing exploitation and exploration strategies, respectively. The BA uses the set of parameters given in Table 1. The pseudo-code of the algorithm is given in Figure 2 and the flow chart of the algorithm is given in Figure 3.

**Table 1:** Basic parameters of the Bees Algorithm.

Parameter	Symbols
Number of scout bees in the selected patches	*n*
Number of best patches in the selected patches	*m*
Number of elite patches in the selected best patches	*e*
Number of recruited bees in the elite patches	*nep*
Number of recruited bees in the non-elite best patches	*nsp*
The size of neighborhood for each patch	*ngh*
Number of iterations	*Maxiter*
Difference between value of the first and last iterations	*diff*

**Figure 4:**
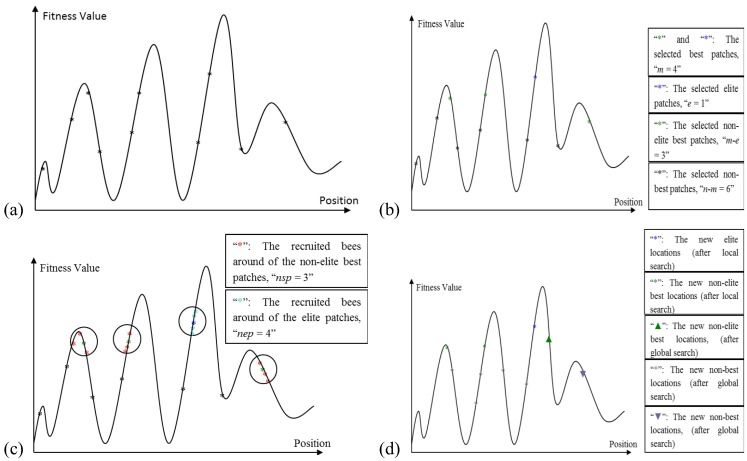
(**a**) The initially selected n patches and their evaluated fitness values; (**b**) Selection of elite and non-elite best patches; (**c**) Recruitment of forager bees to the elite and non-elite best locations; (**d**) Results from basic Bees-inspired Algorithm (BA) after local and global search.

The Algorithm starts with sending *n* scout bees randomly to selected sites (Figure 4a). The fitness values of each site are evaluated and sorted from the highest to the lowest (a maximization problem). The local search step of the algorithm covers the best locations (sites), which are the *m* fittest locations. The *m* best sites are also classified into two sub-groups; elite and non-elite best sites, as given in Figure 4b. The number of elite sites is set as “*e*” and the number of the non-elite best sites is “*m-e*”. The local search process starts with recruiting forager bees in the neighborhood of the best sites. The neighborhood size is set to “*ngh*”. The number of recruited bees in the neighborhood of each elite site is set to “*nep*” and the number of recruited bees in the neighborhood of the non-elite best sites is set to “*nsp*”, as given in Figure 4c. The global search process is a random search process in the *n-m* “non-best” sites, as given in Figure 4d. Finally, the overall locations are sorted according to their fitness value and the process runs until the global optimum is found.

## 5. Improved Bees Algorithm by Adaptive Neighborhood Search and Site Abandonment Strategy

This section describes the proposed improvements to the BA by applying adaptive change to the neighborhood size and site abandonment approach simultaneously. Combined neighborhood size change and site abandonment (NSSA) strategy has been attempted on the BA by Koc [2] who found that the convergence rate of a NSSA-based BA can be slow when the promising locations are far from the current best sites. Here an adaptive neighborhood size change and site abandonment (ANSSA) strategy is proposed which will avoid local minima by changing the neighborhood size adaptively. The ANSSA-based BA possesses both shrinking and enhancement strategies according to the fitness evaluation. The initial move is to implement the shrinking strategy. The strategy works on a best site after a certain number of repetitions. The strategy works until the repetition stops. If, in spite of the shrinking strategy, the number of repetitions still increases for a certain number of iterations, then an enhancement strategy is utilized. Finally, if the number of repetitions still increases for a number of iterations after the use of the enhancement strategy, then that site is abandoned and a new site will be generated. Koc [2] utilized the following parameter for shrinking the neighborhood size and site abandonment strategy: neighborhood size *= ngh*, the shrinking constant = *sc*, the abandoned sites = *aband_site*. In this study four more parameters are introduced. The first is the number of repetitions for each site, denoted as *keep_point*. The *keep_point* records the number of repetitions for all the repetitive results for the best sites. The second parameter is called the “Repetition Number for the Shrinking”, denoted as *rep_nshr*; the number of shrinking is the number of repetitions necessary to start the shrinking strategy, as given in Equations (1) and (2). The third parameter is called “Repetition Number for the Enhancement”, denoted *rep_nenh*. This parameter defines the number of repetitions until the end of the shrinking process, and the beginning of the enhancement process as shown in Equations (1) and (3) [15]. The enhancement process works until the number of the repetitions is equal to the *rep_naban*, which denotes the “Repetition Number for Abandonment Process”. Hence a non-productive site is abandoned and it is stored in *aband_site* list. If there is no better solution than the abandoned site at the end of the searching process, this is the final solution.

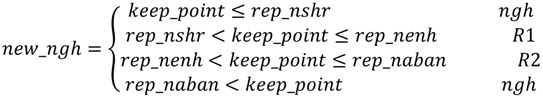
(1)

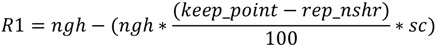
(2)

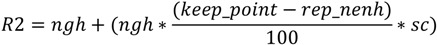
(3)


## 6. Comparison between the ANSSA-Based BA and Other Optimization Methods

### 6.1. Experimental Results

In this sub-section, the ANSSA-based BA was tested on benchmark functions and the results were compared with those obtained using the basic BA and other well-known optimization techniques such as Evolutionary Algorithm (EA), Particle Swarm Optimization (PSO) (standard PSO), Artificial Bee Colony (ABC). There are several differences between these algorithms as given in Section 2. Each algorithm has advantages and disadvantages according to the optimization problems. Therefore there is no perfect algorithm, which works perfectly for all optimization problems [36]. The general weakness and strength of each algorithm used in this study have been summarized below; 

EA has been implemented on several optimization problems, however this algorithm has advantages and disadvantages as given below [37]:

Advantages:
  - Feasibility of finding global optimum for several problems,
  - Availability to combine the hybrid algorithms with EA and others,
  - Implementation with several optimization problems,
  - Availability for real and binary problems.
Disadvantages:
  - Slow convergence rate,
  - Stability and convergence of algorithm is based on recombination and mutation rates,
  - The algorithm may converge to a sub-optimal solution (risk of premature convergence),
  - Algorithm has a weakness on local search,
  - It has a difficult encoding scheme.

PSO has the following advantages and disadvantages [37];

Advantages:
  - The algorithm can easily be implemented;
  - The global search of the algorithm is efficient,
  - The dependency on the initial solution is smaller,
  - It is a fast algorithm,
  - The algorithm has less parameter for tuning.
Disadvantages;
  - The algorithm has a weakness regarding local search,
  - It has a slow convergence rate,
  - It may get trapped in local minima for hard optimization problems.

ABC is also the same as the other algorithms in that it has advantages and disadvantages:

Advantages;
  - The algorithm has strength in both local and global searches [38],
  - Implemented with several optimization problems [38],
Disadvantages;
  - Random initialization,
  - The algorithm has several parameters,
  - Parameters need to be tuned,
  - Probabilistic approach in the local search.

The BA also has advantages and disadvantages compared to the other algorithms [15]:

Advantages:
  - The algorithm has local search and global search ability,
  - Implemented with several optimization problems,
  - Easy to use,
  - Available for hybridization combination with other algorithms.
Disadvantages:
  - Random initialization,
  - The algorithm has several parameters,
  - Parameters need to be tuned.

To analyze the behavior of each algorithm, the benchmark functions have been tested. The ten benchmark functions used for the test are given in Table 2 [38,39]. The basic BA and the enhanced BA require a number of parameters to be set manually. The BA parameters have been empirically tuned and the best combination of the parameter set utilized in this study is given in Table 3 [15]. In addition, the parameters for other optimization algorithms, which are illustrated in this study, are also experimentally tested [38] and are given in Table 4, Table 5, Table 6 and Table 7.

The proposed algorithm was run a hundred times for each function. The performance of the algorithm was assessed according to the accuracy and the average evaluation numbers (Table 8 and Table 9). Experimental results for BA, PSO, EA and ABC were extracted from [39] which were the results of the best performance of each algorithm for the corresponding function.

Further, the t-test was utilized to measure the statistical significance of the proposed algorithm and the basic Bees Algorithm. The results are given in Table 7.

**Table 2:** The selected benchmark functions [38,39].

No	Function Name	Interval	Function	Global Optimum
1	Goldstein &Price (2D)	[−2, 2]	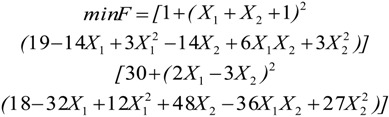	X = [0,−1] F (X) = 3
2	Schwefel (2D)	[−500, 500]	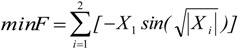	X = [0,0] F(X) = −837.658
3	Schaffer (2D)	[−100, 100]	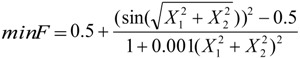	X = (0, 0) F(X) = 0
4	Rosenbrock (10D)	[−1.2, 1.2]	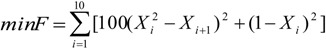	X = [1, 1, 1, 1, 1, 1, 1, 1, 1, 1] F(X) = 0
5	Sphere (10D)	[−5.12, 5.12]	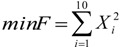	X = [0, 0, 0, 0, 0, 0, 0, 0, 0, 0] F(X) = 0
6	Ackley (10D)	[−32, 32]	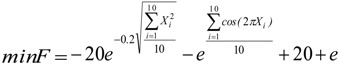	X = [0, 0, 0, 0, 0, 0, 0, 0, 0, 0] F(X) = 0
7	Rastrigin (10D)	[−5.12, 5.12]	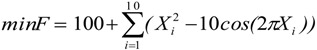	X = [0, 0, 0, 0, 0, 0, 0, 0, 0, 0] F(X) = 0
8	Martin & Gaddy (2D)	[0, 10]	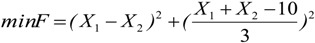	X = [5, 5] F(X) = 0
9	Easom (2D)	[−100, 100]	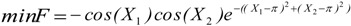	X = [π, π] F(X) = -1
10	Griewank (10D)	[−600, 600]	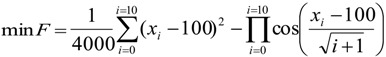	X = [100, 100, 100, 100, 100, 100, 100, 100, 100, 100] F(X) = 0

**Table 3:** The best test parameters for the BA after parameter tuning.

Parameters	Value
Number of Scout Bees in the Selected Patches (*n*)	50
Number of Best Patches in the Selected Patches (*m*)	15
Number of Elite Patches in the Selected Best Patches (*e*)	3
Number of Recruited Bees in the Elite Patches (*nep*)	12
Number of Recruited Bees in the Non-Elite Best Patches (*nsp*)	8
The Size of neighborhood for Each Patches (*ngh*)	1
Number of Iterations (*Maxiter*)	5000
Difference between the First Iteration Value and the Last Iteration (*diff*)	0.001
Shrinking Constant (*sc*)	2
Number of Repetitions for Shrinking Process (*rep_nshr*)	10
Number of Repetitions for Enhancement Process (*rep_nenh*)	25
Number of Repetitions for Site Abandonment (*rep_naban*)	100

**Table 4:** The test parameters for the Evolutionary Algorithms (EA) [38].

Parameters	Crossover	No crossover
Population size	100	
Evaluation cycles (max number)	5000	
Children per generation	99	
Crossover rate	1	0
Mutation rate (variables)	0.05	0.8
Mutation rate (mutation width)	0.05	0.8
Initial mutation interval width α (variables)	0.1	
Initial mutation interval width ρ (mutation width)	0.1	

**Table 5:** The test parameters for the Particle Swarm Optimization (PSO) [38].

Parameters	Value
Population size	100
PSO cycles (max number) *T*	5000
Connectivity	See Table 6
Maximum velocity	See Table 6
C_1_	2
C_2_	2
*w_max_*	0.9
*w_min_*	0.4

**Table 6:** The test parameters for the PSO [38].

Velocity of the each connectivity (Connectivity, *u*)	Max particle velocity *u*			
Connectivity (number of neigbourhood)	(2, 0.005)	(2, 0.001)	(2, 0.05)	(2, 0.1)
	(10, 0.005)	(10, 0.001)	(10, 0.05)	(10, 0.1)
	(20, 0.005)	(20, 0.001)	(20, 0.05)	(20, 0.1)
	(100, 0.005)	(100, 0.001)	(100, 0.05)	(100, 0.1)

**Table 7:** The test parameters for the Artificial Bee Colony (ABC) [38].

Parameters	Value
Population size	100
ABC cycles (max number)	5000
Employed bees n_e_	50
Onlooker bees n_e_	49
Random scouts	1
Stagnation limit for site abandonment *stlim*	50xDimenstion

**Table 8:** Accuracy of the proposed algorithm compared with other well-known optimization techniques.

No.	PSO		EA		ABC		BA		ANSSA-BA	
	Avg. Abs. Dif.	Std. Dev.	Avg. Abs. Dif.	Std. Dev.	Avg. Abs. Dif.	Std. Dev.	Avg. Abs. Dif.	Std. Dev.	Avg. Abs. Dif.	Std. Dev.
1	0.0000	0.0000	0.0000	0.0000	0.0000	0.0001	0.0000	0.0003	0.0000	0.0001
2	4.7376	23.4448	4.7379	23.4448	0.0000	0.0000	0.0000	0.0005	0.0003	0.0007
3	0.0000	0.0000	0.0009	0.0025	0.0000	0.0000	0.0000	0.0003	0.0001	0.0005
4	0.5998	1.0436	61.5213	132.6307	0.0965	0.0880	44.3210	112.2900	0.0000	0.0003
5	0.0000	0.0000	0.0000	0.0000	0.0000	0.0000	0.0000	0.0003	0.0000	0.0000
6	0.0000	0.0000	0.0000	0.0000	0.0000	0.0000	1.2345	0.3135	0.0063	0.0249
7	0.1990	0.4924	2.9616	1.4881	0.0000	0.0000	24.8499	8.3306	0.0002	0.0064
8	0.0000	0.0000	0.0000	0.0000	0.0000	0.0000	0.0000	0.0003	0.0000	0.0000
9	0.0000	0.0000	0.0000	0.0000	0.0000	2.0096	0.0000	0.0003	0.0000	0.0002
10	0.0008	0.0026	0.0210	0.0130	0.0052	0.0078	0.3158	0.1786	0.0728	0.0202

ANNSA: adaptive neighborhood sizes and site abandonment

**Table 9:** Average evaluation of proposed algorithm compared with other well-known optimization techniques.

No.	PSO		EA		ABC		BA		ANSSA-BA	
	Avg. evaluations	Std. Dev.	Avg. evaluations	Std. Dev.	Avg. evaluations	Std. Dev.	Avg. evaluations	Std. Dev.	Avg. evaluations	Std. Dev.
1	3,262	822	2,002	390	2,082	435	504	211	250,049	0
2	84,572	90,373	298,058	149,638	4,750	1,197	1,140	680	250,049	0
3	28,072	21,717	219,376	183,373	21,156	13,714	121,088	174,779	250,049	0
4	492,912	29,381	500,000	0	497,728	16,065	935,000	0	30,893.2	48,267.4
5	171,754	7,732	36,376	2,736	13,114	480	285,039	277,778	25,098.3	36,483.4
6	236,562	9,119	50,344	3,949	18,664	627	910,000	0	234,190.7	54.086.8
7	412,440	67,814	500,000	0	207,486	57,568	885,000	0	93,580	97,429.1
8	1,778	612	1,512	385	1,498	329	600	259	53,005.7	66,284.5
9	16,124	15,942	36,440	28,121	1,542	201	5,280	6,303	250,049	0
10	290,466	74,501	490,792	65,110	357,438	149,129	4,300,000	0	122,713.17	99,163.3

### 6.2. Discussion

In this paper neighborhood search in the BA was investigated. The focus was on improving the BA by utilizing the adaptive neighborhood sizes and site abandonment (ANSSA) strategy. The accuracy of the algorithm was computed with average absolute differences of the best results. According to this, the more accurate results were closer to zero. The proposed algorithm performed significantly better on high dimensional functions. For example, accuracy for 10D-Rastrigin was 0.0002 whereas the accuracy of the basic BA was 24.8499, and for 10D-Ackley it was 0.0063, whereas the accuracy of the basic BA was 1.2345. On another hand the proposed algorithms have less performance on lower dimensional problems, for example the accuracy of the algorithm for 2D-Schwefel function was 0.0003, which was lower than the basic BAs result given in Table 8. This behavior was verified with the number evaluation shown in Table 9 and with the t-test shown in Table 10.

**Table 10:** The statistically significant difference between the adaptive neighborhood sizes and site abandonment in (ANSSA)-based BA and the basic BA.

No.	Function	Significance between the basic BA and the improved BA	
		Significant ( α < 0.05)	α
1	Goldstein & Price (2D)	No	0.200
2	Schwefel (2D)	No	0.468
3	Schaffer (2D)	No	0.801
4	Rosenbrock (10D)	No	0.358
5	Sphere (10D)	No	0.433
6	Ackley (10D)	Yes	0.020
7	Rastrigin (10D)	Yes	0.007
8	Martin & Gaddy (2D)	No	0.358
9	Easom (2D)	No	0.563
10	Griewank (10D)	Yes	0.020

As it is shown in Table 9, the average numbers of evaluations for 10D-Rastrigin and 10D-Ackley were found with the proposed algorithm to be 93,580 and 234,190.7, respectively, whereas the basic BA results were 885,000 and 910,000 respectively. However, the number of evaluations for low dimensional functions was higher than the number of evaluations received from the basic BA for same functions. With respect to the “no free lunch” theorem [36], if an algorithm performs well on a certain class of problems then it necessarily pays for that with degraded performance on the set of all remaining problems. From the results it can be clearly seen that the algorithm’s performance on 10D functions is better than on 2D ones. Moreover, this behavior is evident when comparing the enhanced BA with other optimization algorithms. The better performance of the proposed algorithm on the higher dimensional functions can be attributed to its adaptive response during the neighborhood search. However, this adaptive response may increase computational time for lower dimensional functions. For this reason, the proposed algorithm is expected to have a better performance for the higher dimensional problems, which need more computational time.

A t-test was carried out on the results obtained for the basic BA and the improved BA in order to see if there was any significant difference between the performances of the two methods. This was done by looking for evidence for the rejection of the null hypothesis *i.e.*, no significant difference between the performances of the two algorithms. An α value of less than 0.05 in Table 10 indicates when the improved BA was significantly better than the basic BA. The t-test results in Table 10 show that the enhanced BA performed better than the basic BA for higher order functions and that they were similar for the lower order functions.

## 7. Conclusions

In this paper, an optimization algorithm inspired by the natural foraging behavior of honey bees, called the Bees Algorithm, has been discussed, and an enhanced version called ANSSA-based Bees Algorithm has been proposed.

The proposed ANSSA-based has been successfully applied on continuous type benchmark functions and compared with other well-known optimization techniques. To test the performance of the proposed algorithm, the following comparison approaches have been utilized: accuracy analysis, average evaluation and t-test. According to the results of the accuracy analysis and the average evaluation, the proposed algorithm performed better on higher dimensional than lower dimensional functions.

Finally, the statistical significance of proposed algorithm has been computed with a t-test and the results were compared with the basic Bees Algorithm. Based on the t-test results it can be concluded that the results of the proposed algorithm are statistically significant than the results of basic Bees Algorithm. Thus the proposed algorithm performed better than the basic Bees Algorithm on higher dimensional functions such as, Ackley (10D) and Griwank (10D). 
